# Examining trabecular morphology and chemical composition of peri-scaffold osseointegrated bone

**DOI:** 10.1186/s13018-020-01931-z

**Published:** 2020-09-14

**Authors:** Linwei Lyu, Shicai Yang, Ye Jing, Chunqiu Zhang, Jikun Wang

**Affiliations:** 1 Tianjin Key Laboratory for Advanced Mechatronic System Design and Intelligent Control, Tianjin University of Technology, No. 391 Binshui Xidao, Xiqing District, Tianjin, China; 2 National Demonstration Center for Experimental Mechanical and Electrical Engineering Education (Tianjin University of Technology), Tianjin, China; 3 Second Affiliated Hospital of Tianjin University of TCM, Tianjin, China; 4 Institute of Automatic Control and Robotics, Faculty of Mechatronics, Warsaw University of Technology, Warsaw, Poland

**Keywords:** Osseointegration, Bone ingrowth, Porous titanium scaffold, Preload, Bone defect

## Abstract

**Background:**

Porous titanium alloy scaffold fabricated by 3D printing technology could induce osseointegration well to repair bone defect during early postoperative period. However, trabecular histomorphological features and chemical compositions of ingrowth bone in the long term after surgery still lacked in-depth research.

**Methods:**

Fourteen New Zealand rabbits were divided into two groups (7 rabbits in surgery group and 7 rabbits in control group). A 3D-printed porous titanium alloy scaffold was implanted into right femoral condyle of each rabbit in the surgery group. Preload was produced at the surface between bone tissue and scaffold through interference assembly during implantation process. Rabbits in the control group were feed free. All rabbits were sacrificed to extract femoral condyles at week 12 after surgery. All right femoral condyles were performed micro-CT scanning to test bone mineral density (BMD) and trabecular histomorphological parameters, including bone volume fraction (BV/TV), bone surface/volume ratio (BS/BV), bone surface density (BS/TV), structure model index (SMI), trabecular thickness (Tb.Th), trabecular number (Tb.N), trabecular separation (Tb.Sp), porosity (PO), connectivity density (Conn.Dn), and degree of anisotropy (DA). Scanning electron microscope was used to observe osteogenesis peri-scaffold. Fourier transform infrared spectroscopy (FTIR) scanning was performed to analyze chemical compositions of peri-scaffold trabeculae. All trabecular morphological parameters and BMDs were statistically analyzed between surgery group and control group.

**Results:**

The pores of scaffold were filled with ingrowth bone tissues after 12 weeks osseointegration. However, the mean BMD peri-scaffold in surgery group was 800 ± 20 mg/cm^3^, which was 18.37% lower than that in the control group. There was a significant decrease in BV/TV, Tb.N, and BS/TV, and there was a significant increase in Tb.Sp and PO between the surgery group and control group (*p* < 0.05). There were no significant differences in Tb.Th, SMI, Conn.Dn, BS/BV, and DA. Although ingrowth of bone tissue was very effective, some fragmented connective tissues were still found instead of bone tissues on the partial beams of scaffolds through SEM images. It was found from FTIR that there was no significant hydroxyapatite peak signal in surgery group. Collagen in the control group mainly existed as cross-link structure, while non-cross-link structure in the surgery group.

**Conclusions:**

Preload could promote the same good osseointegration ability as chemical surface modification method in the early term after surgery, and better osseointegration effect than chemical surface modification method in the mid-long term after surgery. However, histomorphological features of peri-scaffold trabeculae were still in deterioration and low collagen maturity caused by stress shielding. It was suggested from this study that extra physical training should be taken to stimulate the bone remodeling process for recovering to a healthy level.

## Introduction

Large bone defects caused by trauma and bone tumor cannot heal by itself although rehabilitation mechanism existed in bone tissue [1]. Autologous bone graft was considered the golden repair material for treating bone defect [2]. At present, with the introduction of 3D printing technology, porous titanium scaffolds with similar micro-structural features and mechanical properties could be manufactured in accordance with defect dimensions [3–5]. Osteoblast migration was induced to perform osteogenesis differentiation closely integrated on the inner and outer surfaces of scaffolds after implantation [6]. This process of bone ingrowth without interposed soft tissue was called osseointegration. 3D printing technology could reduce elastic modulus of porous titanium scaffold significantly to fit bone tissue. General elastic modulus and compressive strength of cancellous bone have been reported in the range of 2–5 GPa and 11–24 MPa, respectively [7–10]. However, elastic modulus and compressive strength of scaffolds with porosity of 70% were still in the range of 5.7–6.7 GPa and 74–155 MPa, respectively [11]. Therefore, stress shielding would change mechanical micro-environment of bone tissue and hinder bone remodeling process. In the long-term after surgery, osteolysis might occur in the region of stress shielding resulting in the risk of secondary fracture [12–14].

To address this issue, there were several mechanical stimulations improving bone ingrowth [15, 16]. In vitro and vivo observations of osseointegration showed that mechanical stimulation had a promising effect on bone ingrowth. Jemt et al. found that bone-to-metal contact of good fit implant without preload was about 40% after 3 weeks osseointegration [15]. With the increasing of preload, bone-to-metal in high preload group was significantly increased compared with no preload group. Matsuzaki et al. performed finite element analysis and related experiments to compare stress distribution between preload contact and bonded contact [16]. In the preload contact model, stress was more widely distributed than bonded contact. External static load bearing exercises were performed on individuals with transfemoral amputation fitted with an osseointegrated implant to facilitate bone remodeling [17]. From clinical treatment effect, kinematic ability and weight-bearing ability of individuals were better than that of untrained individuals.

Bone ingrowth could be induced into scaffold by preload. The strength of ingrowth bone still should be considered to avoid risk of secondary fracture. The strength of bone was determined by both quantity and quality [18]. Factors determining bone quantity was bone mineral density (BMD). BMD was the strongest index affecting bone strength and the single strongest predictor of fracture risk non-invasive. Dual energy X-ray was widely used in clinical to test mean BMD to predict fracture risk. Micro-CT was used in conjunction with hydroxyapatite calibration phantom to accurately measure BMD in vitro. Meanwhile, micro-structure features of peri-scaffold trabeculae were also measured. Factors that determined bone quality referred to chemical composition and trabecular morphological features. Fourier transform infrared spectroscopic (FTIR) imaging could provide information on chemical composition and collagen structure of bone tissues. In a bone spectrum, PO_4_ area (910–1184 cm^−1^) corresponded to the symmetric and antisymmetric stretching of the phosphates, and CO_3_ area (810-860 cm^−1^) corresponded to the symmetrical stretching of the carbonates [19]. Collagen maturity referred to collagen cross-link structure expressed in peak from 1660 to 1690 cm^−1^ [20]. These indicators might help to study bone quality and fracture risk in more detail. The objective of this study was to examine the effect of preload on osseointegrated bone quantity and quality around 3D-printed titanium porous scaffold after 12 weeks implantation. The trabecular histomorphology features and chemical compositions of ingrowth bone peri-scaffold were scanned through Fourier transform infrared spectroscopy and micro-CT scanning. We wished to find out the promoting effect of preload implantation of 3D-printed titanium porous scaffold on bone remodeling process peri-scaffold.

## Materials and methods

### Scaffolds preparation

3D-printed porous titanium alloy scaffolds were specifically designed for New Zealand rabbit femoral condyles. Cylindrical scaffolds with a diameter of 2 mm and a length of 5 mm were fabricated using Arcam EBM Q10plus electron beam machine. The other manufacturing parameters were with a porosity of 70%, a pore diameter of 0.65 mm and a wire diameter of 0.32 mm. All scaffolds were soaked into iodophor for disinfection until the surgeries. The scaffolds were cleaned in demineralized water and then air-dried for implantation after anesthesia of rabbits.

### Implantation surgery procedure

**Fig. 1:**
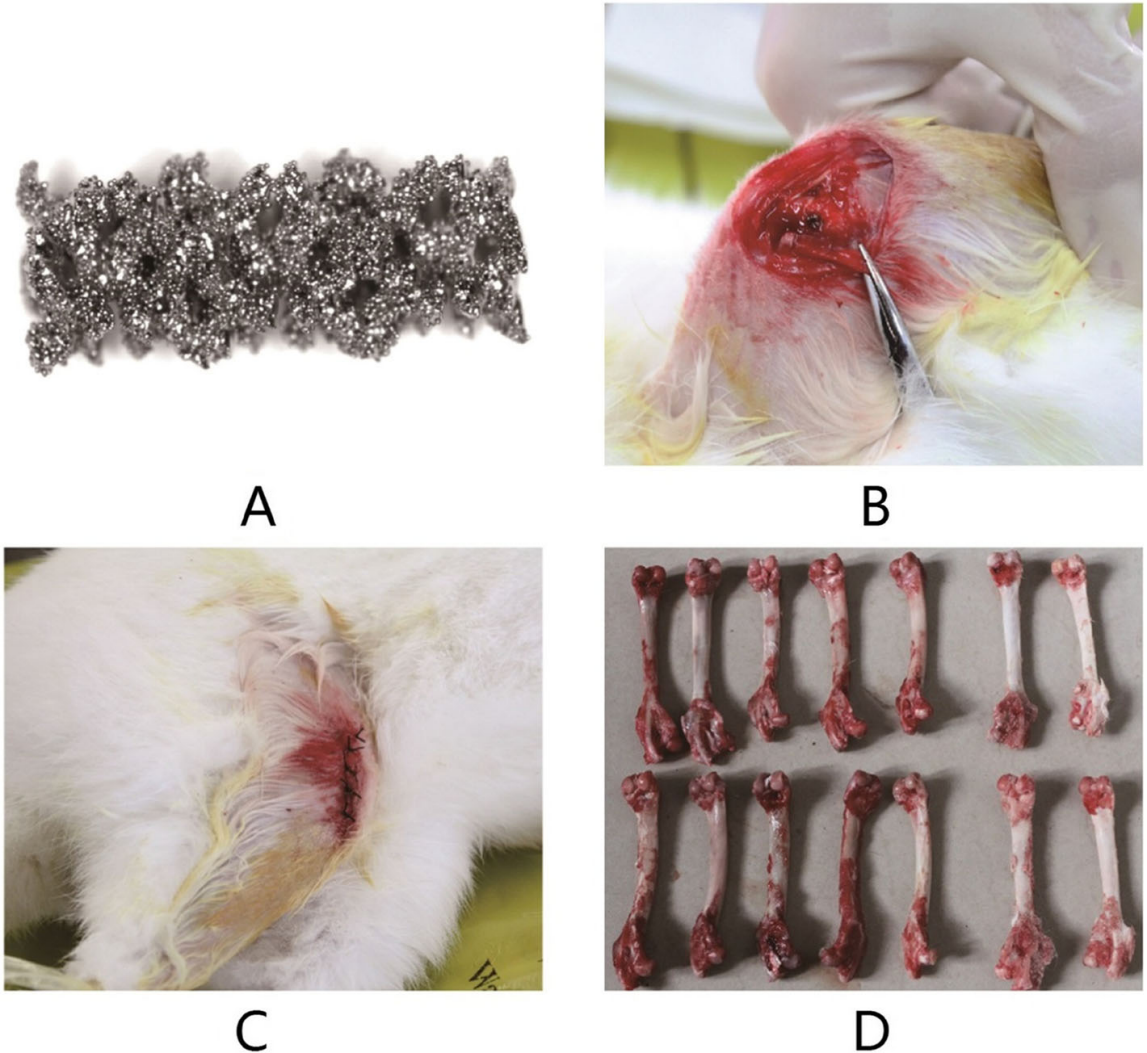
Scaffold implantation surgery process of a rabbit. **a** A 3D-printed porous titanium scaffold. **b** Surgery process of implantation. **c** The stitching process after surgery. **d** All right femurs from rabbits after 12 weeks osseointegration

Rabbits’ surgery procedures were conducted according to “China Guidelines for treating laboratory animals well”. The study procedure was approved by the Animal Ethics Committee of the Tianjin institute of Pharmaceutical Science with the serial number of IMPS-EAEP-Z-2019108-01. Fourteen New Zealand rabbits (2.5 months old, average weight of 2.5 kg, license number SCXK(Beijing) 2016-0007) were divided into 2 groups (7 in surgery group and 7 in control group) for experiments. Rabbits in the surgery group were operated under anesthesia performed by intraperitoneal injection of chloral hydrate solution (10%w/v, 3.5 ml/kg). Right femoral condyles were shaved clearly to expose skins. Lidocaine hydrochloride (2 ml, 0.02 g/ml) was injected subcutaneously at knee joint for local anesthesia after skin disinfection with iodine. An incision of 4–5 cm was made at lateral knee to exposed femoral condyle. A small amount of lidocaine hydrochloride would be sprayed while rabbits were waking up from anesthesia. A bone defect with a diameter of 1.9 mm and a depth of 5 mm was drilled on the lateral femoral condyle through the cortical and trabecular bone. The cavity was thoroughly rinsed with physiological saline solution after drilling. A 3D-printed porous titanium alloy scaffold was implanted into drilling hole with preload produced by interference assembly. The diameter of scaffold was 2 mm, and the cavity at rabbit femoral condyle was drilled using bench drill with self-manufactured 1.9-mm-diameter drill. The scaffold was implanted into the cavity slowly using compression function of material testing machine. A range of 0.001–0.002 strain at the edge of cavity caused by was detected using digital image correlation system during multiple in vitro implantation preliminary experiments. According to bone remodeling theory, this strain range belonged to light overload, which could promote bone remodeling. The implantation process was shown in Fig. 1. The incision was sutured and disinfected with iodide. After waking up, rabbits were injected with enrofloxacin (0.2 ml/kg) into the muscle of the operative leg for 3 days. Post-surgical pain was controlled by intramuscular injection of buprenorphine. After the surgical procedures, the rabbits were confined to cages and maintained with a regular laboratory diet. All rabbits were sacrificed by air embolism after 12 weeks healing surgery. The right femoral condyles containing scaffolds were dissected and immediately frozen in a − 80 °C freezer [21, 22]. Considering thermal expansion and cold contraction issue on the samples in the low temperature storage, we performed a thermal calculation on porous titanium scaffold during the process of low temperature freeze from 20 to − 80 °C. The main chemical compositions of trabecular bone and scaffold were hydroxyapatite and titanium alloy (Ti6Al4V), respectively. The linear thermal expansion coefficient of hydroxyapatite and titanium alloy were 1.15 × 10^−5^/°C [23] and 1.17 × 10^−5^/°C [24], respectively. The radius of titanium alloy scaffold was 2.5 mm. Therefore, the cold contraction dimension of trabecular bone and scaffold on the interface along radial direction from 20 °C to − 80 °C were all about 2.9 *μ*m. The equations were solved as follows:

$$ 2500\mu m\times \left[20-\left(-80\right)\right]\times 1.15\times {10}^{-5}=2.875\mu m $$

$$ 2500\kern0.28em \mu \mathrm{m}\times \left[20-\left(-80\right)\right]\times 1.17\times {10}^{-5}=2.925\kern0.28em \mu \mathrm{m} $$

In our study, mean trabecular thickness of rabbit femoral condyle was about 345 μm. Therefore, the effect of cold contractions between hydroxyapatite and titanium alloy was weak. Because the resolution of femoral micro-CT image was about 32 μm, the deformations of our sample could not be detected either.

### Histology and histomorphometry

All samples from surgery group and control group were scanned by micro-CT with a pixel size of 32 μm, a segmentation thickness of 32 μm, a region of interest of 1500 × 1500 pixels, voltage of 79 kV, and current of 125 μA. Meanwhile, a bone density calibration phantom (QRM micro-CT HA Phantom, QRM Gmbh) was scanned with the same scanning parameters to calculate BMD. All micro-CT images were imported into CTAn (short for CT-analyser, Bruker Gmbh), which was a morphometry and densitometry measurement accessory provided by Bruker skyscan 1076 micro-CT workstation, to calculate trabecular morphological parameters around scaffolds, including bone volume fraction (BV/TV), bone surface/volume ratio (BS/BV), bone surface density (BS/TV), structure model index (SMI), trabecular thickness (Tb.Th), trabecular number (Tb.N), trabecular separation (Tb.Sp), porosity (PO), connectivity density (Conn.Dn), and degree of anisotropy (DA), as well as bone mineral density (BMD). Trabecular morphological parameters and BMD of trabecular bone were expressed as an average ± standard deviation. Independent-sample *t* tests were performed on each trabecular morphological parameter and BMD in SPSS 17.0 (IBM INC.) with significance level of 95%.

**Fig. 2:**
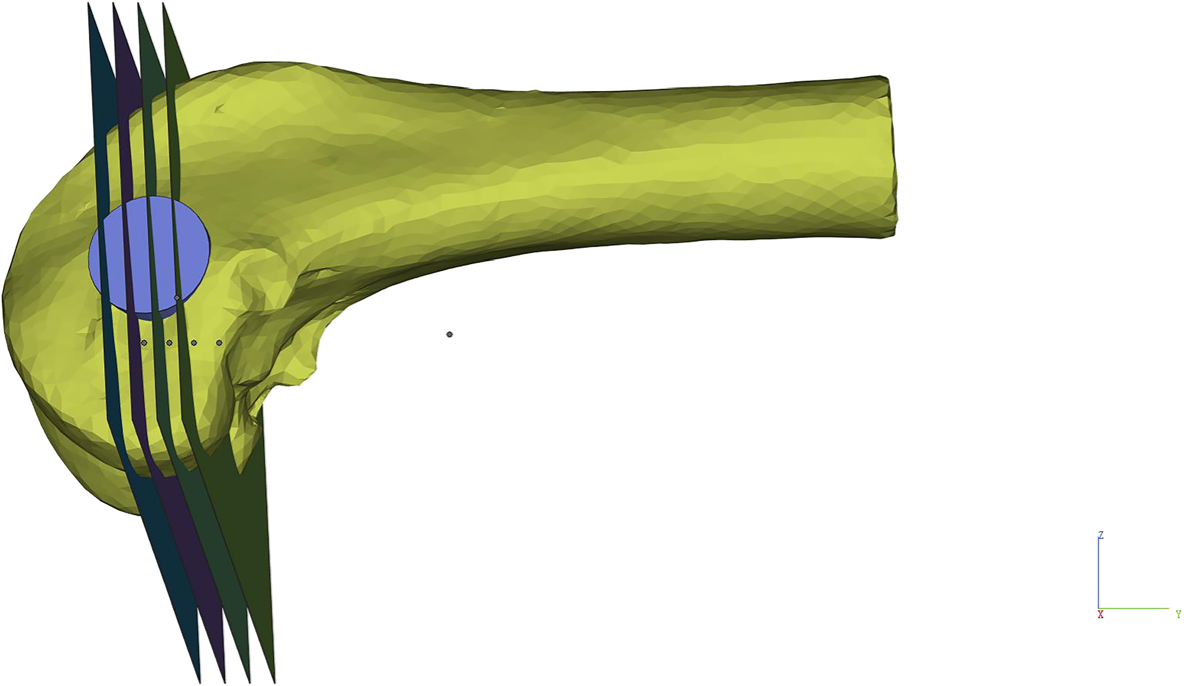
Hard tissue section method for rabbit femoral condyles with titanium alloy scaffolds

Femoral condyles containing scaffolds in surgery group were performed undecalcificated bones slicing (E300CP, EXAKT1 Apparatebau GmbH & Co., Norderstedt, Germany) with the slice thickness of 1 mm. There were total four slices obtained from each femoral condyle. The slicing method was shown in Fig. 2. Femoral condyles in control group were sliced at the same place.

Slice 1 and slice 2 were performed SEM (SU8100, HITACHI Inc.) analysis. The bone-scaffold interfaces were evaluated for each femoral condyle sample. Each section was digitized (× 30, × 200, × 400, × 2000) to evaluate bone ingrowth on the beam of scaffold. Slice 3 and slice 4 were performed FTIR (Nicolet 5700, Thermo Fisher Inc.) scanning on bone tissue inside scaffolds. Scanning parameters were times of 256, scanning range of 2000 cm^−1^ and 650 cm^−1^ and scanning resolution of 4 cm^−1^.

## Results

Mean BMDs in control group and surgery group were calculated as 980 ± 90 mg/cm^3^ and 800 ± 20 mg/cm^3^, respectively, after 12 weeks healing. Our statistical results found that BMD in surgery group decreased 18.37% (*p* < 0.05) compared with that in the control group. Trabecular morphological parameters were shown in Table 1. There were significant differences in BV/TV, Tb.N, Tb.Sp, PO, and BS/TV (*p* < 0.05) between the surgery group and control group. No significant differences were shown in Tb.Th, Conn.Dn, DA, BS/BV, and SMI between the surgery group and control group.

**Table 1:** Trabecular morphological parameters and BMDs of rabbit femoral condyles between the surgery group and control group

Groups	BV/TV (-)	BS/BV (1/pixel)	BS/TV (1/pixel)	SMI (-)	Tb.Th (pixel)	
**Control group**	0.1596 ± 0.01796	0.3975 ± 0.0472	0.0624 ± 0.0067	0.2783 ± 0.2578	9.7824 ± 1.1867	
**Surgery group**	0.1338 ± 0.0087	0.3803 ± 0.0869	0.0498 ± 0.0034	0.3570 ± 0.1908	10.7757 ± 0.8328	
**Difference test**	**0.012**	0.703	**0.003**	0.575	0.137	
**Groups**	**Tb.N (1/pixel)**	**Tb.Sp (pixel)**	**PO (-)**	**Conn.Dn (1/pixel^3)**	**DA (-)**	**BMD(mg/cm**^**3**^**)**
**Control group**	0.0163 ± 0.0024	99.0811 ± 11.5257	0.8263 ± 0.0122	0.00008	1.9607 ± 0.0714	980 ± 90
**Surgery group**	0.0133 ± 0.0010	113.9774 ± 7.9093	0.8511 ± 0.0202	0.0009	1.9025 ± 0.0954	800 ± 20
**Difference test**	**0.048**	**0.032**	**0.041**	0.673	0.291	**0.001**

*p* < 0.05

**Fig. 3:**
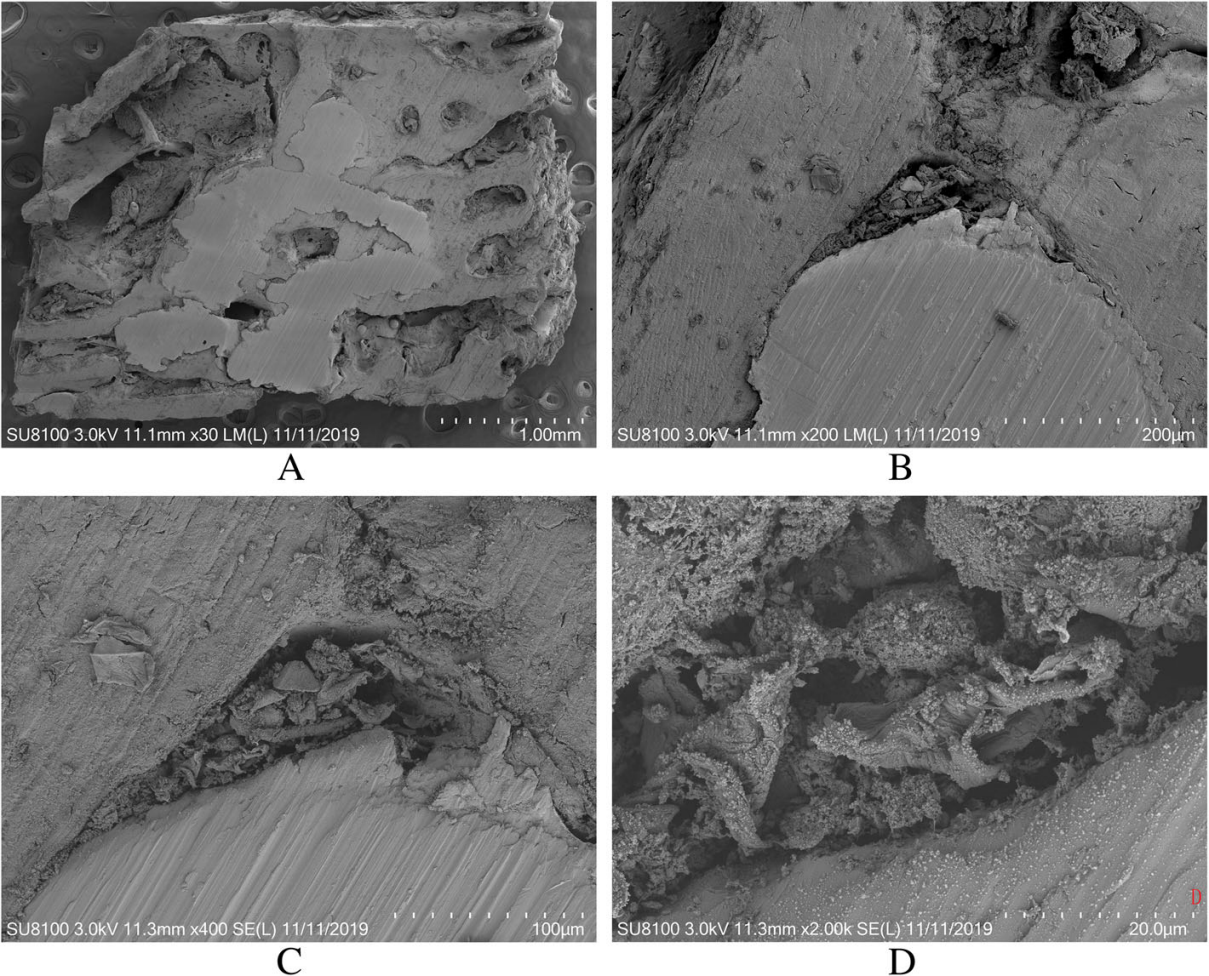
Typical SEM images from osseointegration region of a rabbit femoral condyle. The magnifications of the images from **a** to **d** were × 30, × 200, × 400, and × 2000, respectively

Four slices with titanium alloy scaffolds were obtained using hard tissue slicer. Slice 1 and slice 2 were performed SEM scanning. Typical SEM images of rabbit femoral condyles from surgery group are shown in Fig. 3. Some connective tissues were found at the surface of partial beams inside the scaffolds.

**Fig. 4:**
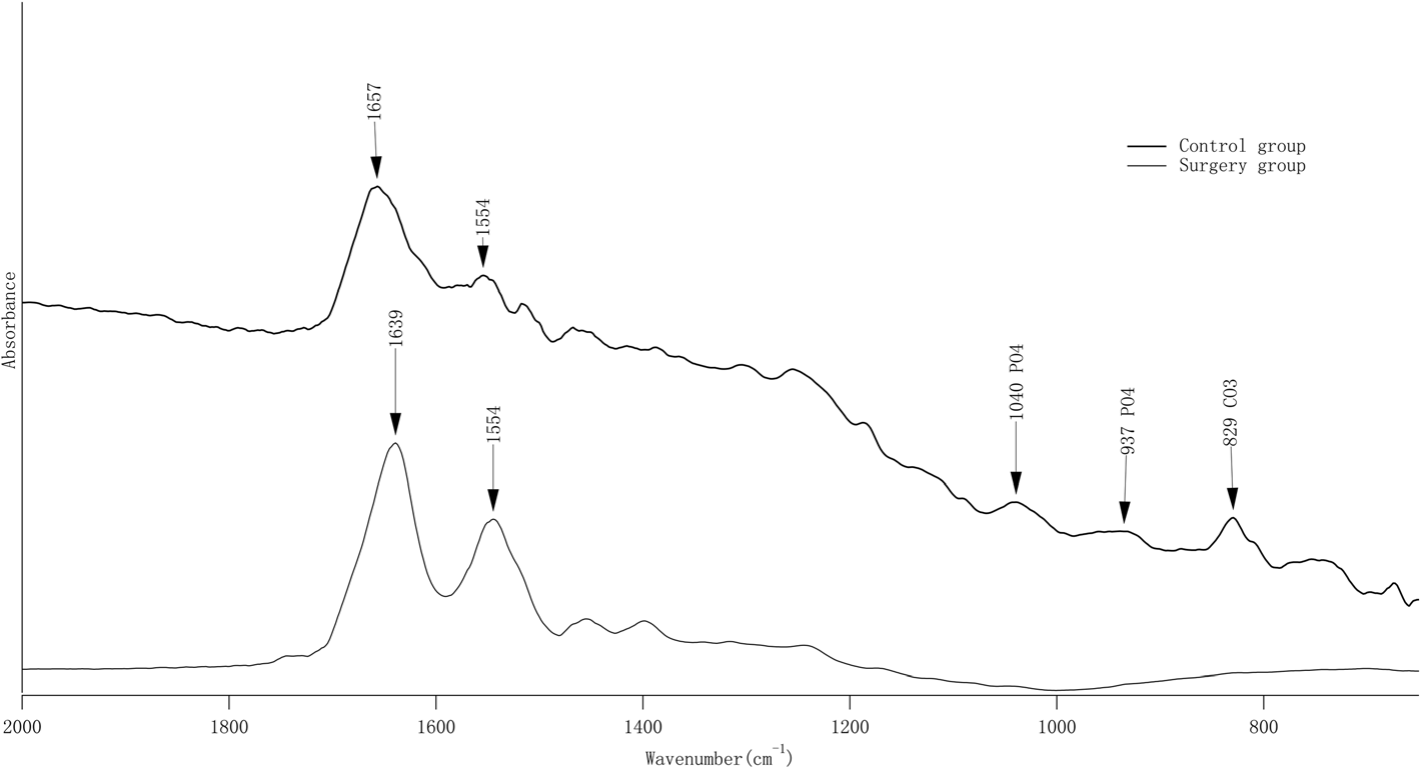
Typical FTIR spectrum of ingrowth bone tissue in the control group and surgery group

Slice 3 and slice 4 were performed FTIR scanning. Typical spectrums of bone tissues from the control group and surgery group were shown in Fig. 4. The secondary structure of collagen from samples with scaffolds revealed peak at 1639 cm^−1^, while the collagen peak in control group was mainly in 1657 cm^−1^. There was no distinct hydroxyapatite peak or very low signal existed in 500–650 cm^−1^ and 900–1200 cm^−1^ in surgery group.

## Discussion

This study was designed to evaluate osseointegration of 3D printed porous titanium alloy scaffolds implanted with preload. Preload produced by interference fit at interface between scaffold and bone tissue could recover mechanical micro-environment and stimulate the bone remodeling process. Compared with current widely used surface chemical modification method, the bone ingrowth effect of our designed interference assembly implantation method was similar to that coating with hydroxyapatite.

The effect of osseointegration depended not only on the ingrowth bone mass, but also on the trabecular micro-structure and chemical compositions. In our previous study, BMD was found to be a significant indicator of trabecular strength at macro level. There were still other factors that could determine bone strength, such as trabecular morphological parameters at a microlevel and chemical compositions at a nanolevel.

BMD was a mainly non-invasive test to evaluate bone strength. Micro-CT HA phantom was scanned together with bone tissues to calibrate mean BMD around scaffolds. In our study, the strength of ingrowth bone tissue was not as strong as control samples. The risk of secondary fractures might exist in the mid-long term after surgery. Chalisserry el al. used nano-hydroxyapatite scaffolds to repair rabbit femoral condyle defect. After 8 weeks healing, BMD in scaffold region was 800 ± 10 mg/cm^3^ [25]. This result was consistent with ours. The scaffold coated with nano-hydroxyapatite was regarded as good osteoinduction, which could promote osseointegration effectively. Our scaffolds without any coating were exerted on the preload during the implantation process. The similar effect of bone ingrowth was found compared with coating scaffolds. If both methods were all applied to bone defect repair, the effect of bone ingrowth would be better. However, it was revealed that scaffolds with hydroxyapatite coating cause bone tissue deterioration in the long-term healing after surgery. Yamasaki el al. found that BMD started to decrease after 5 weeks and BMD decreased about 5–10% at week 12 compared with that at week 5. BMD declined even more dramatically after 12–48 weeks [26]. Zhang et al. found BMD reached the highest at week 12 of osseointegration [27]. Our experiments only last to week 12, and no continuous BMD measurements were taken after surgery. Therefore, BMD in our experiments did not show significant deterioration trend in accordance with data from Chalisserry. Meanwhile, we cannot confirm that BMD in surgery group reached peak at week 12. Surface chemical modification could only induce osteogenesis in the early postoperative period, continuous bone remodeling still required appropriate mechanical micro-environment.

**Fig. 5:**
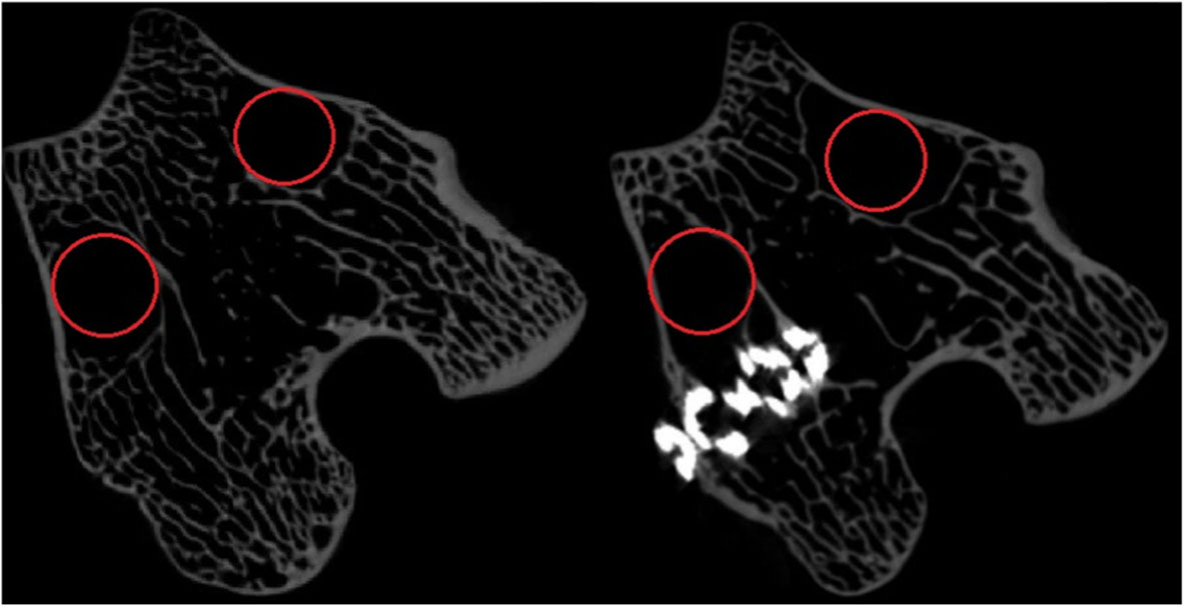
Typical trabecular features observation between the control group and surgery group. The left image was from the control group and the right image was from the surgery group. The regions with red circles in the right image marked the larger holes compared with the left image

Trabecular morphology was another indicator of bone strength. There were no significant differences in BS/BV, SMI, Tb.Th, Conn.Dn, and DA between surgery group and control group. It was concluded from these results that trabecular bone was mainly plate-like structure and the main growth direction of trabeculae had not changed. However, BS/TV in surgery group decreased significantly in surgery group, which meant trabecular surface area (BS) in surgery group decreased significantly. Ossification on the scaffolds could not significantly increase trabecular surface area, but trabecular resorption under stress shielding reduced trabecular surface area significantly. Meanwhile, BS/BV did not change significantly. We believed that bone volume (BV) also decreased significantly in surgery group, so BS/BV did not change significantly. This result could be verified by the significant increase of Tb.Sp and PO. Tb.Sp was defined as mean degree of separation, and PO was defined as porosity of trabeculae. Tb.Sp in surgery group was significantly higher than control group. We randomly extracted two micro-CT images from each group to verify this result, which was shown in Fig. 5. Trabecular number (Tb.N) per unit volume in surgery group was found significantly less than control group. We could conclude that the introduction of scaffold destroyed initial mechanical environment hindering normal bone remodeling process. Moreover, some non-connected trabeculae might be produced during bone defect drilling process. Non-connected trabeculae and stress shielding trabeculae caused by titanium alloy scaffolds could not receive loading signal and then osteolysis followed. In summary, the promotion of preload on osseointegration was positive; however, the stress shielding of scaffolds also required attentions. In our previous study, the strength of our 3D-printed porous titanium scaffolds were about 600 MPa, but still significantly higher than surrounding trabeculae to cause bone resorption and osteolysis.

In order to observe ingrowth bone tissue in more detail, we performed SEM scanning to observe histomorphology of peri-scaffold bone. There were some connective tissues found on partial beam of the scaffolds. This kind of tissue had similar image features in micro-CT with trabeculae, but mechanical property was worse. This might influence mean BMD value. Some researchers had found connective tissue existed in the interface between bone tissue and porous structure inside the scaffold. Marloes et al. found that non-load control group showed connective tissues on the scaffold beam structure surface after 12 weeks osseointegration [28]. Samples in experiment group were all observed to have new bone formation. Internal spaces of scaffolds were all in the range of stress shielding because of far higher strength than bone tissue. Preload had definite effect on peri-scaffold bone tissue, but very little effect on bone tissue inside the scaffold. Fan et al. found that there were some connective tissues inside the pore of titanium scaffold; meanwhile, good osseointegration effects were observed at the surface of scaffold pores. It was shown that maturity and strength of bone tissue at surface of scaffold were higher than that inside the scaffold during new bone ingrowth process [29]. We also found some fragmented connective tissue inside the scaffold. Chemical composition analysis would provide information about this kind of tissue more precisely. Chemical composition of bone tissue could reflect bone maturity through collagen cross-link form [18, 30]. We performed FTIR scanning to analyze test chemical composition of ingrowth bone tissue from surgery group and bone tissue in the same area from control group. The FTIR spectrum revealed that chemical composition of ingrowth bone was quite different from that from the control group. The spectrums showed that the peak of collagen transferred from 1657 in the control group to 1639 cm^−1^ in the surgery group. It was concluded that collagen inside the scaffold might be in non-cross-link structure, and collagen from control group was mainly in cross-link structure. We could not find peak of PO_4_ (1040 cm^−1^–937 cm^−1^) from the surgery group. However, a distinct peak of PO_4_ was found in the control group. This also suggested that the mineralization of ingrowth bone was poor at week 12.

Therefore, the improvement of osseointegration in the long-term after surgery must require additional rehabilitation training. The external load could be transferred from ingrowth bone tissue to the inside of the scaffold to stimulate bone remodeling process inside the scaffold to achieve perfect osseointegration.

Some limitations existed in this study. First, positive control group was absent from the experiments. The contributions of preload, self-healing and porous titanium alloy induction on osseointegration promotion could not be quantitatively analyzed according to the existed results although large bone defect could not heal completely on their own. The current osseointegration was a join effect. In the following experiments, bone defect group without scaffold and surgery group without preload will be added. Second, interference fit error existed in the implantation surgeries. It was found that 0.1-mm interference would produce about 300 μƐ at the surface of bone defect through finite element analyses. However, drilling errors and beam fractures of scaffolds would cause strain errors at the surface of bone defect. Further investigations will determine the range of interference that could promote osseointegration best. Third, we only investigated osseointegration response 12 weeks after surgery, which was inadequate to determine whether the enhanced bone ingrowth will remain further. Therefore, the osseointegration effect of preload at 16 weeks and 20 weeks after surgery will be added to the experiments for comparison analysis. Fourth, we only performed qualitative analysis of FTIR currently, while quantitative analysis would bring us more information on chemical compositions of ingrowth bone.

## Conclusion

It was concluded from the findings of this study that the ability of preload to promote osseointegration was consistent with scaffold surface chemical modification method at 12 weeks after surgery. However, parts of scaffold beam were covered by some connective tissues rather than bone tissues. Bone tissues around the scaffolds were unable to provide sufficient strength as in the control group for load bearing. There were also plenty of immature regenerated new bone. Mechanical stimulation for bone defect area had a great significance to mid-long-term recovery after surgery.

## Data Availability

Data and materials presented in this paper can be shared upon request.

## Abbreviations

BMD: Bone mineral density
FTIR: Fourier transform infrared spectroscopic
BV/TV: Bone volume fraction
BS/BV: Bone surface/volume ratio
BS/TV: Bone surface density
SMI: Structure model index
Tb.Th: Trabecular thickness
Tb.N: Trabecular number
Tb.Sp: Trabecular separation
PO: Porosity
Conn.Dn: Connectivity density
DA: Degree of anisotropy
SEM: Scanning electron microscope
BS: Trabecular surface area
BV: Bone volume
